# FGFR3 Alterations and Nectin-4 Expression as Therapeutic Biomarkers in Bladder Cancer: A Systematic Review and Single-Arm Meta-Analysis

**DOI:** 10.3390/ijms27115007

**Published:** 2026-06-01

**Authors:** Petar Antonov, Gabriela Raycheva, Denis Eshrefov, Angel Belov, Petar Uchikov, Atanas Ivanov, Veselin Popov, Matteo Pacini, Alessandro Zucchi, Andrea Nicolini, Plamen Penchev

**Affiliations:** 1 Department of Urology and General Medicine, Medical University of Plovdiv, 4002 Plovdiv, Bulgaria; denis.eshrefov@mu-plovdiv.bg (D.E.); angel.belov@mu-plovdiv.bg (A.B.); atanas.ivanov@mu-plovdiv.bg (A.I.); 2 Department of Clinical Oncology, Medical University of Plovdiv, 4002 Plovdiv, Bulgaria; gabriela.raycheva@mu-plovdiv.bg (G.R.); dr.v_popov@yahoo.com (V.P.); 3 Department of Special Surgery, Medical University of Plovdiv, 4002 Plovdiv, Bulgaria; 4 Department of Translational Research and New Technologies in Medicine and Surgery, University of Pisa, 56126 Pisa, Italy; matteopacini93@gmail.com (M.P.); zucchi.urologia@gmail.com (A.Z.); 5 Department of Clinical and Experimental Medicine, University of Pisa, 56126 Pisa, Italy; 6 Faculty of Medicine, Medical University of Plovdiv, 4002 Plovdiv, Bulgaria; sonaonetrick@abv.bg

**Keywords:** bladder cancer, FGFR3, nectin-4, biomarkers, personalized medicine

## Abstract

Bladder cancer is a molecularly heterogeneous malignancy in which biomarker-driven therapies increasingly shape clinical management. Fibroblast growth factor receptor 3 (FGFR3) alterations and nectin-4 expression are key therapeutic targets, yet their integrated biological and clinical relevance remains unclear. A systematic search of PubMed, Scopus, and Cochrane Central was conducted from database inception to 22 February 2026 (PROSPERO: CRD420261309413). Studies reporting the prevalence of FGFR3 alterations and/or nectin-4 expression in bladder cancer were included. Proportions were pooled using a random-effects model with restricted maximum likelihood and Freeman–Tukey transformation. Heterogeneity was assessed with I^2^ and Cochran’s Q. Fourteen studies (three randomized and 11 observational), including 3955 patients (mean age: 67.34 years), were analyzed. The pooled prevalence of FGFR3 alterations was 52% (95% CI: 23.33–80.12; I^2^ = 99%), while that of nectin-4 expression was 78% (95% CI: 64.23–89.81; I^2^ = 91%). FGFR3 prevalence varied significantly by disease stage, study design, and region, with higher rates in advanced/metastatic disease and randomized trials (*p* < 0.05). Nectin-4 expression was generally high across included studies, although interpretation was limited by the small number of studies and assay variability. Sensitivity analyses showed the stability of estimates; however, interpretation is limited by substantial heterogeneity. The observed prevalence estimates are strongly influenced by study design, biomarker selection, and assay variability, limiting their interpretation as true biological prevalence. These results should, therefore, be interpreted cautiously and viewed as descriptive rather than definitive estimates. Separate analyses of biomarker-enriched trials and unselected cohorts are necessary to obtain clinically meaningful estimates.

## 1. Introduction

Bladder cancer is a molecularly heterogeneous malignancy characterized by distinct genomic and phenotypic alterations that shape tumor biology, clinical behavior, and therapeutic response [1,2]. Advances in molecular profiling have enabled the identification of actionable targets and the development of biomarker-driven treatment strategies, particularly in urothelial carcinoma [3]. Among the most clinically relevant alterations, fibroblast growth factor receptor 3 (FGFR3) mutations and fusions are frequently observed, especially in luminal molecular subtypes, and represent clinically actionable biomarkers in specific therapeutic contexts, particularly in FGFR-targeted therapies [3,4,5]. Concurrently, nectin-4, a transmembrane cell adhesion molecule encoded by PVRL4, is highly expressed in urothelial carcinoma and serves as the therapeutic target of the antibody–drug conjugate enfortumab vedotin [6,7,8,9]. Beyond their individual predictive roles, both biomarkers are implicated in key oncogenic processes, including cell proliferation, adhesion, and tumor microenvironment interactions, underscoring their biological and therapeutic significance. The prevalence and biological role of these biomarkers vary substantially across disease stages (non-muscle invasive, muscle-invasive, and metastatic disease), which complicates the interpretation of pooled estimates across heterogeneous populations.

Despite these advances, the current body of evidence remains fragmented. FGFR3 alterations and nectin-4 expression have largely been investigated in isolation, limiting insight into their potential biological interplay and combined clinical relevance. Data on their co-expression patterns and associations with molecular subtypes remain limited and inconsistently reported. Moreover, the lack of integrated analyses hampers understanding of how these biomarkers may jointly influence therapeutic response, resistance mechanisms, and optimal treatment sequencing in the era of targeted therapies and antibody–drug conjugates. Variability in biomarker assessment methodologies and heterogeneity across study designs further complicate cross-study comparisons and translational interpretation [10,11]. Consequently, a comprehensive and quantitatively synthesized evaluation of these biomarkers is lacking.

To address this gap, we conducted a systematic review and single-arm meta-analysis to provide an integrated assessment of FGFR3 alterations and nectin-4 expression in bladder cancer. This study aims to summarize the reported prevalence patterns and variability in FGFR3 alterations and nectin-4 expression across different study contexts.

## 2. Methods

### 2.1. Eligibility Criteria

This systematic review and meta-analysis followed the Cochrane Handbook for Systematic Reviews of Interventions and the Preferred Reporting Items for Systematic Reviews and Meta-Analysis Statement [12,13]. This meta-analysis did not require Institutional Review Board approval because it used data from previously published and publicly available articles. Studies that met all the following criteria were included in the meta-analysis: (1) studies including adult patients (≥18 years) diagnosed with histologically confirmed bladder cancer or urothelial carcinoma (studies including mixed urothelial carcinoma populations were eligible only if bladder-specific data could be independently extracted); (2) studies including reported data on at least one of the following molecular markers: FGFR3 alterations (including mutations, fusions, amplification, or overexpression) and nectin-4 expression (assessed via immunohistochemistry (IHC), RNA expression, or molecular profiling); (3) studies whose outcomes of interest included the prevalence of FGFR3 alterations and the prevalence of nectin-4 expression levels; and (4) observational studies (retrospective or prospective), cohort studies, translational molecular studies, clinical trials reporting biomarker data, cohort studies, and case–control studies. Given the variability in biomarker definitions across studies, FGFR3 alterations (mutations, fusions, amplification, and overexpression) and nectin-4 expression (assessed via different platforms and thresholds) were grouped broadly; however, this heterogeneity is recognized as a major limitation. Studies were excluded if they met one of the following criteria: (1) studies on pediatric populations (<18 years), and studies focusing exclusively on upper tract urothelial carcinoma without separable bladder data or non-urothelial histologies (e.g., pure squamous-cell carcinoma or adenocarcinoma); (2) studies not reporting extractable prevalence or expression data, or studies evaluating biomarkers unrelated to FGFR3 or nectin-4 without subgroup data; (3) studies not reporting at least one of the outcomes of interest; (4) studies with overlapping populations; and (5) case reports or case series, narrative reviews, systematic reviews and meta-analyses, editorials, letters, dissertations, gray literature, conference abstracts without full-text availability, and animal studies. This systematic review and meta-analysis was registered with the International Prospective Register of Systematic Reviews (PROSPERO) under the ID “CRD420261309413”.

### 2.2. Search Strategy and Data Extraction

We systematically searched PubMed, Scopus, and Cochrane Central from database inception to 22 February 2026, with the following search strategy: (“Urinary Bladder Neoplasms” [Mesh] OR “Non-Muscle Invasive Bladder Neoplasms” [Mesh] OR “bladder cancer” OR “urothelial carcinoma” OR “urothelial cancer” OR NMIBC OR MIBC OR “non-muscle invasive bladder cancer” OR “muscle-invasive bladder cancer” OR “muscle-invasive bladder neoplasm”) AND (“Receptor, Fibroblast Growth Factor, Type 3” [Mesh] OR FGFR3 OR “FGFR3 mutation” OR “FGFR3 alteration” OR Nectin-4 OR NECTIN4 OR “Nectin-4 expression” OR “Nectin-4 antigen”). All included studies were verified to be publicly available at the time of search execution. Restrictions were applied to only English-language articles. Gray literature was excluded. We manually searched the references of all included studies to identify any additional studies. Two authors (D. E. and A. B.) independently extracted the data using predefined search criteria, quality assessment methods, and the Rayyan software (https://www.rayyan.ai; access on 20 March 2026) [14]. Any disagreements between these authors were resolved via consensus.

### 2.3. Endpoints and Subgroup Analyses

The meta-analysis included FGFR3 alteration and NECTIN4 expression endpoints. Additionally, we conducted a subgroup analysis based on tumor stage, risk-of-bias assessment, study design, and geographic region. Given the known selection bias in biomarker-enriched clinical trials, analyses were stratified a priori by study design into (1) biomarker-enriched interventional trials and (2) unselected observational cohorts. Pooled prevalence estimates from these groups were interpreted separately and not considered representative of the same biological population. We acknowledge that randomized controlled trials may include biomarker-enriched populations and, therefore, may not reflect general prevalence. Their inclusion was intended to capture the full spectrum of reported data, but the results were interpreted with caution.

### 2.4. Quality Assessment

For non-randomized studies, the risk of bias was assessed using the Cochrane Collaboration’s tool for assessing the risk of bias in non-randomized studies of interventions (ROBINS-I) [15]. The ROBINS-I tool categorizes the risk of bias as low, moderate, serious, or critical. The risk of bias for the RCTs was assessed using the Cochrane Collaboration’s tool for assessing the risk of bias in randomized trials (RoB 2) [16]. The RoB 2 tool categorizes the risk of bias as low, some concerns, or high. Two authors (D.E. and A.B.) independently performed the assessments, resolving disagreements via consensus. Publication bias was evaluated using contour-enhanced funnel plots with the trim-and-fill method, which allows for a better interpretation of asymmetry related to statistical significance thresholds, in line with the recommendations by Nakagawa et al. (2017) [17]. Additional methods, such as p-curve or p-uniform analysis, were not feasible due to the absence of reported exact *p*-values or test statistics in all included studies. Following the Cochrane guidelines, the Egger test was not performed because fewer than 10 studies were included in the meta-analysis [18].

### 2.5. Statistical Analysis

Proportions with 95% confidence intervals (CIs) were computed for all outcomes of interest. The Freeman–Tukey double arcsine transformation was applied before pooling to stabilize variance in the presence of proportions close to 0 or 1 [18]. Pooled estimates were back-transformed to the original proportion scale for interpretation. A random-effects model was used to account for heterogeneity across studies, employing the restricted maximum-likelihood estimator. Heterogeneity was assessed using the I^2^ statistic and Cochran’s Q test. Two-sided *p*-values < 0.05 were considered statistically significant. Subgroup analyses were performed based on risk-of-bias assessment, tumor stage, study design, and geographic region to minimize the risk of selection bias. Subgroup differences were assessed using the Q-test for heterogeneity between groups. Leave-one-out sensitivity analyses (LOO) were conducted to evaluate the robustness of the findings. A Baujat plot was generated to identify studies contributing most to heterogeneity and their influence on the overall meta-analysis results. This diagnostic tool visually represents the balance between a study’s contribution to heterogeneity (x-axis) and its weight in the meta-analysis (y-axis), aiding in the interpretation of outlier or highly influential studies. Statistical analyses were performed using the R software, version 4.3.1, with the packages “metafor” and “meta” [19]. Given the anticipated high heterogeneity, pooled estimates were considered descriptive summaries rather than precise estimates of a single underlying population parameter.

## 3. Results

### 3.1. Study Selection and Baseline Characteristics

**Figure 1:**
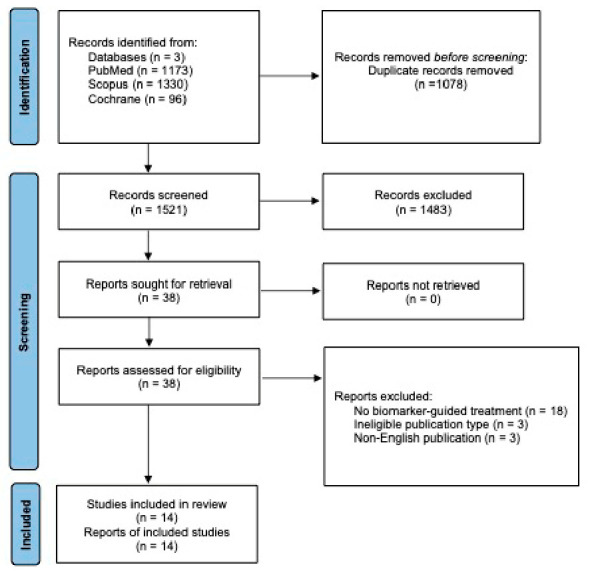
PRISMA flowchart and study selection.

The search strategy yielded a total of 2926 results. After removing duplicate records and unrelated articles or abstracts, the remaining 38 studies were fully reviewed to determine whether they met the inclusion and exclusion criteria (Figure 1). Fourteen studies (three RCTs and eleven observational) were included, with 3955 patients [10,11,20,21,22,23,24,25,26,27,28,29,30,31]. The mean age of the population was 67.34 years. Males accounted for 70%. The study characteristics are presented in Table 1 and Table 2.

**Table 1:** Baseline characteristics of the included studies.

Study/Characteristics	Design	Level of Evidence	Geographic Region	No. of Patients	FGFR3 alt	Nectin-4 Exp.	Age *	Males %	Race	ECOG Status	Metastases	Tumor Stage
Guercio et al. 2023 [30]	Prospective observational	2b	Multicountry	1030	274	N/A	67.8	65	Caucasian; Non-Hispanic: 90%	NR	NR	Non-muscle-invasive
Miyake et al. 2025 [27]	Multimodal translational	2b	Japan and USA	70	N/A	67	67	N/A	NR	NR	NR	Muscle-invasive
Catto et al. 2024 [20]	Randomized controlled trial	1b	Multicountry	882	336	N/A	69	76	White: 55%; Asian: 25%; NR: 20%	0–80% 1–20%	NR	Non-muscle-invasive
Loriot Y. et al. 2023 [22]	Randomized controlled trial	1b	Multicountry	177	177	N/A	66	70	White: 60%; Asian: 27%	0–46% 1–43% 2–9%	74%	Advanced/metastatic
Siefker-Radtke et al. 2024 [21]	Randomized controlled trial	1b	Multicountry	264	264	N/A	67	81	White: 54%; Asian: 21%; NR: 22%	0–51% 1–42% 2–6%	67%	Advanced/metastatic
Ueki H. et al. 2022 [11]	Retrospective observational	2b	Japan	23	N/A	18	68	78	Asian	0.1–83% 2.3–17%	57%	Advanced/metastatic
Gupta et al. 2025 [25]	Retrospective observational	2b	USA	1048	176	N/A	72	71	White: 76%; Other: 12%	0–40% 1–41% 2–18%	NR	Advanced/metastatic
Teo et al. 2020 [26]	Retrospective observational	2b	USA	151	23	N/A	67	70	NR	0–80% 1–20%	70%	Muscle-invasive
Rose et al. 2021 [29]	Retrospective observational	2b	USA	88	17	N/A	72	60	White: 76%; Black: 18%	0–23% 1–35% 2–24%	60%	Advanced/metastatic
Reverdy et al. 2025 [24]	Retrospective observational	2b	France	142	47	N/A	71	74	White	0.1–88% 2–12%	48%	Advanced/metastatic
Hsueh et al. 2025 [23]	Retrospective observational	2b	Taiwan	37	N/A	25	63	50	Asian	0–25% 1–70% 2–5%	55%	Advanced/metastatic
Klumper N. et al. 2023 [31]	Retrospective observational	2b	Germany	137	NR	110	68	65	NR	NR	NR	Muscle-invasive
Necchi A. et al. 2024 [10]	Prospective observational	2b	Multicountry	173	134	NR	69	77	White: 62%; Asian: 12%; NR: 22%	0–34% 1–52% 2–13%	70%	Advanced/metastatic
Olah et al. 2025 [28]	Retrospective observational	2b	Multicountry	23	NR	18	69	77	NR	NR	5%	Muscle-invasive

Footnotes: *—Mean age; N/A—not applicable; NR—not reported.

**Table 2:** Biomarker assessment methods, alteration/expression definitions, and key findings of included studies.

Study (Author, Year)	Study Design	N	Population	Treatment Context	Biomarker	Assessment Method	Key Findings
Gupta et al., 2025 [25]	Real-world clinicogenomic cohort	1048	mUC	ICPI/chemotherapy	FGFR3; TMB	Hybrid-capture NGS/CGP	FGFR3 alone not predictive; FGFR3 + high TMB may predict ICPI benefit
Teo et al., 2020 [26]	Retrospective cohort analysis	151	MIBC/mUC	Platinum-based chemotherapy	FGFR3	MSK-IMPACT NGS; TCGA genomic data	FGFR3 alteration linked to poorer NAC response; no clear impact in mUC
Rose et al., 2021 [29]	Retrospective	88	mUC	ICPI	FGFR3	DNA and RNA sequencing	FGFR3 alterations did not predict ICB response or survival
Reverdy et al., 2025 [24]	Multicenter retrospective cohort	142	mUC	First-line platinum-based chemotherapy ± ICI	FGFR3	NGS/local genomic testing	FGFR3 status did not predict PFS, OS, or ORR with platinum chemotherapy
Necchi et al., 2024 [10]	Phase II single-arm trial	173	Unresectable/mUC	Pemigatinib	FGFR3	Genomic testing	Pemigatinib showed modest activity in FGFR3-altered UC
Siefker-Radtke et al., 2024 [21]	Phase III RCT	264	mUC	Erdafitinib vs. pembrolizumab	FGFR3	Central/local genomic testing	Erdafitinib improved ORR but not OS vs. pembrolizumab
Guercio et al., 2023 [30]	Real-world clinicogenomic cohort	1030	UC across disease states	Erdafitinib/ICI	FGFR2/3	MSK-IMPACT NGS; cfDNA MSK-ACCESS	FGFR3 varied by disease state; erdafitinib ORR: 40%; short PFS
Catto et al., 2024 [20]	Phase II randomized trial	882	High-risk NMIBC	Erdafitinib vs. intravesical chemotherapy	FGFR3/2	Genomic testing	Erdafitinib improved RFS vs. intravesical chemotherapy
Loriot et al., 2023 [22]	Phase III RCT	177	mUC	Erdafitinib vs. chemotherapy	FGFR3	Central genomic testing	Erdafitinib improved OS and PFS vs. chemotherapy
Hsueh et al., 2025 [23]	Retrospective	37	mUC	First-line GC/Gcarbo	Nectin-4	Immunohistochemistry (H-score)	High nectin-4 showed trend toward better PFS/OS, especially with GC
Klümper et al., 2023 [31]	Retrospective	137	UC with matched metastases; EV-treated mUC	Enfortumab vedotin	Nectin-4	IHC (membranous expression; H-score)	Low/absent nectin-4 associated with EV resistance and shorter PFS
Oláh et al., 2025 [28]	Retrospective	23	MIBC	Perioperative platinum/EV relevance	Nectin-4	IHC; membranous H-score	Nectin-4 varied by subtype; no OS association; low expression linked to greater platinum benefit
Miyake et al., 2025 [27]	Translational cohort study	70	MIBC	Chemotherapy/EV resistance	Nectin-4	IHC; RNA-seq; RT-PCR/Western blot	Chemotherapy downregulated nectin-4; low nectin-4/basal subtype linked to worse prognosis
Ueki et al., 2022 [11]	Retrospective cohort	23	Advanced/metastatic UC	Pembrolizumab after chemotherapy	Nectin-4	IHC; nectin-4 H-score/intensity	Strong nectin-4 expression associated with higher DCR

### 3.2. Pooled Analyses of the Included Studies

#### 3.2.1. FGFR3 Alteration

**Figure 2:**
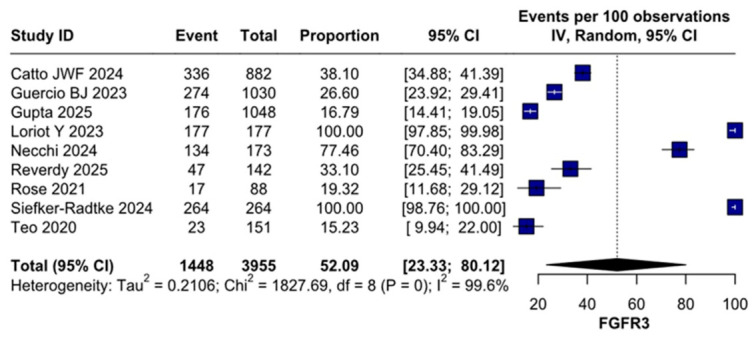
Forest plot of the pooled proportion of FGFR3 alterations across nine studies, showing a summary prevalence of 52.09% (95% CI: 23.33–80.12). Random-effects model using REML estimator with Freeman–Tukey transformation [10,20,21,22,24,25,26,29,30].

**Figure 3:**
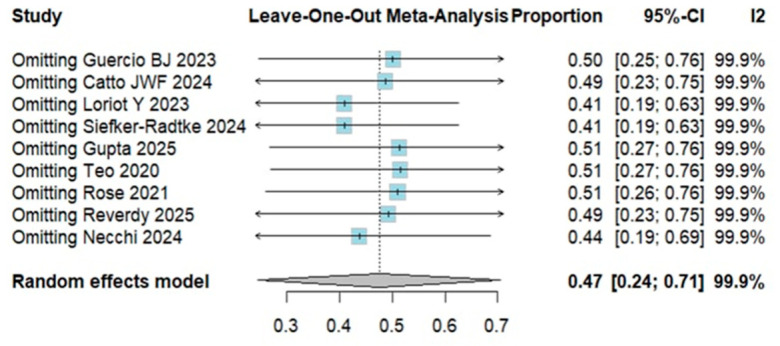
LOO sensitivity analysis for FGFR3 alteration. The results demonstrate a stable pooled effect size across all iterations, although heterogeneity remained high throughout all studies. Random-effects model using REML estimator with Freeman–Tukey transformation [10,20,21,22,24,25,26,29,30].

**Figure 4:**
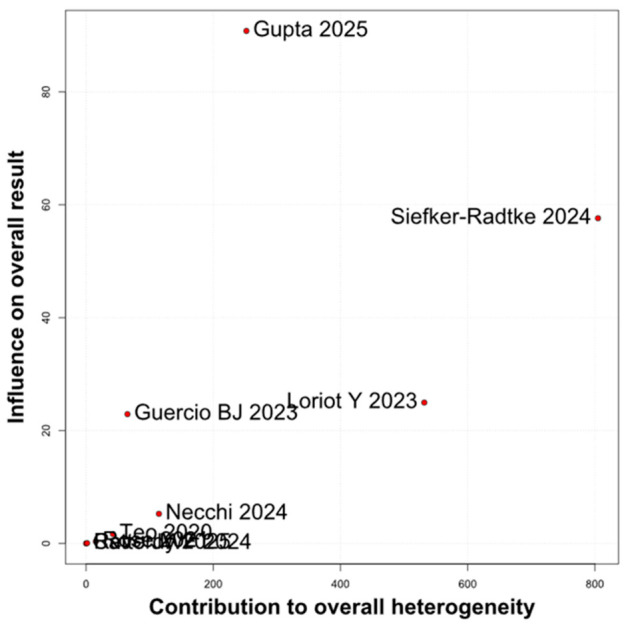
Baujat plot of study influence for FGFR3 alteration. The plot identifies the studies by Gupta (2025) [25] and Siefker-Radtke (2024) [21] as the most influential, significantly contributing to both the overall pooled effect and the observed heterogeneity [10,20,21,22,24,25,26,29,30].

The pooled estimate of 52% reflects a mixture of biomarker-enriched trials and unselected cohorts and should not be interpreted as a true population-level prevalence (Figure 2). Given the very high heterogeneity, these pooled estimates should be interpreted cautiously and do not represent a single biologically homogeneous population. The LOO analysis revealed that the overall effect size was consistent across sensitivity analyses but subject to substantial heterogeneity (Figure 3). This suggests that no single study had a disproportional influence on the overall outcome. The Baujat plot identified the studies by Gupta 2025 et al. [25] and Siefker-Radtke 2024 et al. [21]. as potentially influential, contributing substantially to the overall result and heterogeneity (Figure 4).

#### 3.2.2. Narrative Synthesis

Given the extreme heterogeneity (I^2^ > 90%), pooled estimates were complemented by a narrative synthesis. Observational studies generally reported lower FGFR3 prevalence (approximately 15–40%), whereas biomarker-enriched trials reported substantially higher rates (>80%), reflecting selection criteria rather than biological differences. For nectin-4, expression remained consistently high across studies, although variability in assay methods and thresholds contributed to uncertainty.

#### 3.2.3. Nectin-4 Expression

**Figure 5:**
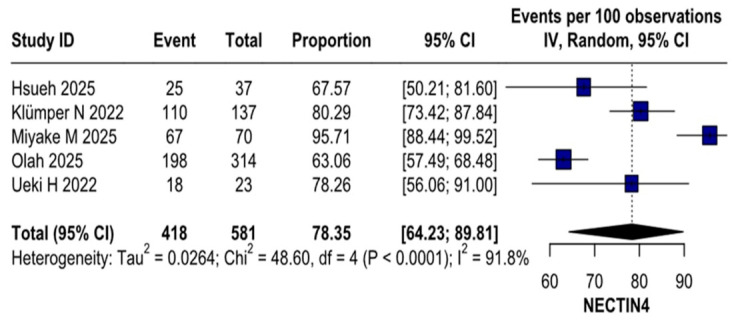
Forest plot of the pooled proportion of nectin-4 expression across five studies, showing a summary prevalence of 78.35% (95% CI: 64.23–89.81). Random-effects model using REML estimator with Freeman–Tukey transformation [11,23,27,28,31].

**Figure 6:**
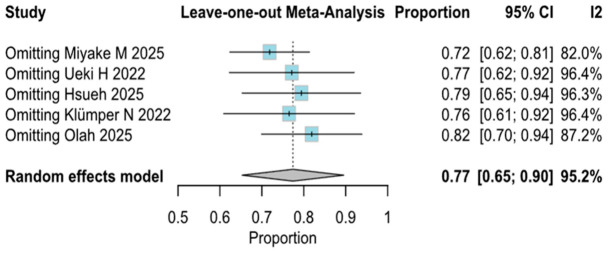
LOO sensitivity analysis for nectin-4 expression. The results were consistent across all iterations; however, heterogeneity remained high throughout all studies. Random-effects model using REML estimator with Freeman–Tukey transformation [11,23,27,28,31].

**Figure 7:**
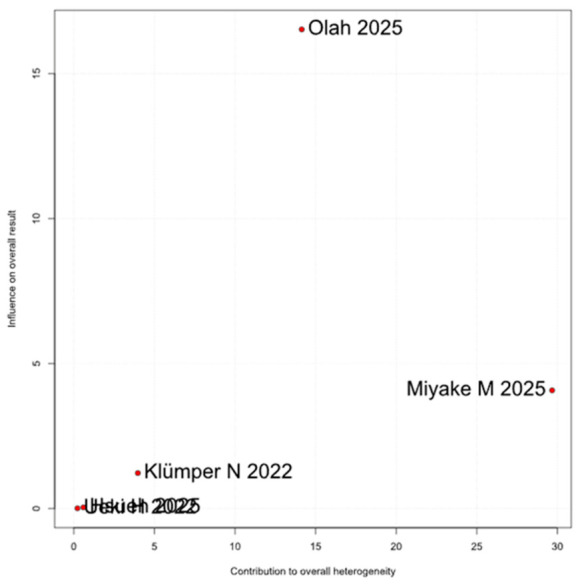
Baujat plot of study influence for nectin-4 expression. The plot identifies the studies by Olah (2025) [28] and Miyake M. (2025) [27] as the most influential, contributing significantly to both the overall pooled effect and the observed heterogeneity [11,23,27,28,31].

Only five studies contributed to this analysis, limiting statistical power and the reliability of subgroup analyses. The pooled estimate of 78% reflects a small number of heterogeneous cohorts and should not be interpreted as a true population-level prevalence (Figure 5). Although fewer biomarker-enriched trials were included for nectin-4, variability in study design and patient selection may still contribute to the observed heterogeneity. Given the very high heterogeneity, these pooled estimates should be interpreted cautiously and do not represent a single biologically homogeneous population. The LOO analysis showed the overall effect size was consistent across sensitivity analyses but subject to substantial heterogeneity (Figure 6). This suggests that no single study has a disproportional influence on the overall outcome. The Baujat plot identified the studies by Olah (2025) [28] and Miyake M. (2025) [27] as potentially influential, contributing substantially to the overall result and heterogeneity (Figure 7).

### 3.3. Subgroup Analyses

Reported subgroup *p*-values refer to the Q-test for between-subgroup heterogeneity.

#### 3.3.1. Tumor Stage

##### FGFR3

**Figure 8:**
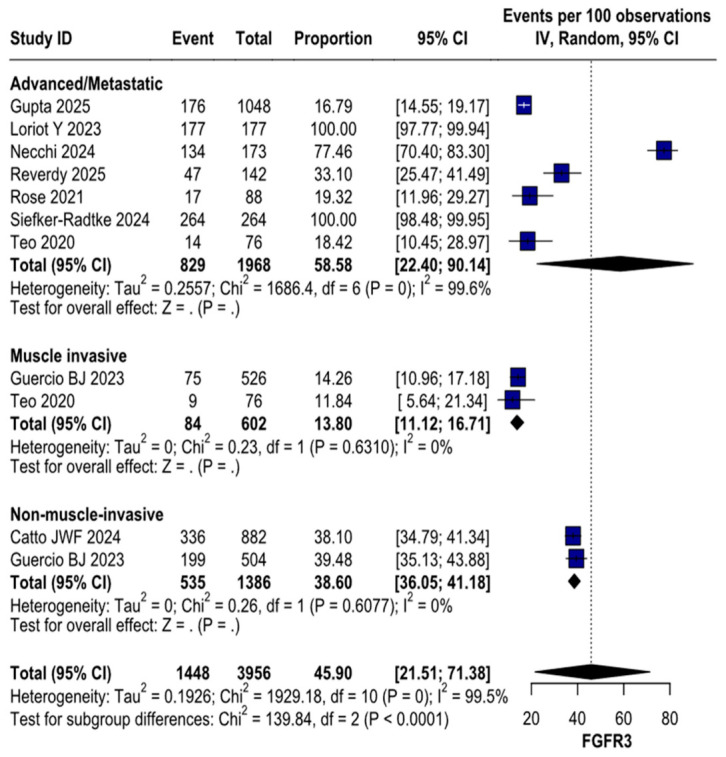
Subgroup analysis of FGFR3 prevalence by disease stage. Significant differences were observed across cohorts (*p* < 0.0001), with the highest prevalence in the advanced/metastatic group (58.58%; 95% CI: [22.40, 90.14]). Heterogeneity was substantial in the advanced subgroup (I2 = 99.6%) but absent in the MIBC and NMIBC groups (I2 = 0%). Random-effects model using REML estimator with Freeman–Tukey transformation [10,20,21,22,24,25,26,29,30].

Subgroup analysis revealed significant differences in FGFR3 prevalence across disease stages (*p* < 0.0001). The pooled proportion was highest in the advanced/metastatic group (58.58%; 95% CI: 22.40–90.14%), followed by the non-muscle-invasive (38.60%; 95% CI: 36.05–41.18%) and muscle-invasive (13.80%; 95% CI: 11.12–16.71%) groups. Heterogeneity was high in the advanced subgroup (I2 = 99.6%) but absent in both the muscle-invasive and non-muscle-invasive cohorts (I2 = 0%) (Figure 8).

##### Nectin-4

**Figure 9:**
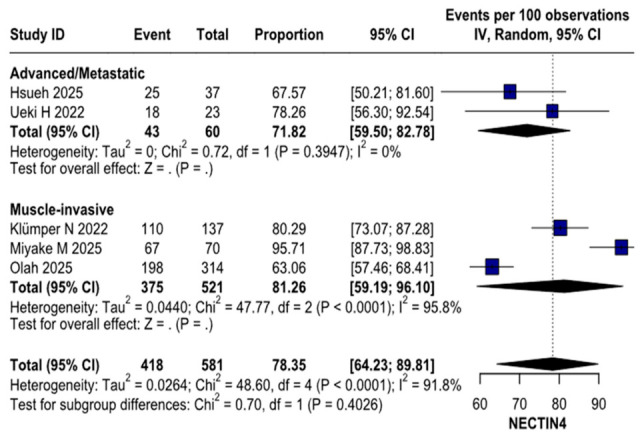
Subgroup analysis of nectin-4 prevalence by disease stage. No significant difference was found between cohorts (*p* = 0.4026), with pooled proportions of 81.26% for MIBC and 71.82% for advanced/metastatic disease. Heterogeneity was absent in the metastatic group (I2 = 0%) but remained high in the muscle-invasive cohort (I2 = 95.8%). Random-effects model using REML estimator with Freeman–Tukey transformation [11,23,27,28,31].

The subgroup analysis revealed no significant difference in nectin-4 prevalence between disease stages (*p* = 0.4026). The pooled proportion was 81.26% (95% CI: 59.19–96.10%) for the muscle-invasive cohort and 71.82% (95% CI: 59.50–82.78%) for the advanced/metastatic cohort. Heterogeneity was absent in the metastatic group (I2 = 0%) but remained high among muscle-invasive studies (I2 = 95.8%) (Figure 9).

#### 3.3.2. Study Design

##### FGFR3

**Figure 10:**
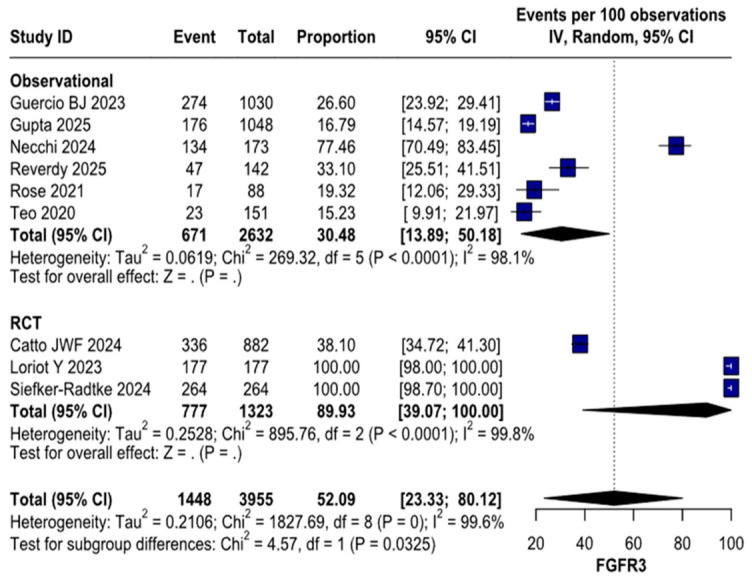
Subgroup analysis of FGFR3 prevalence by study design. A significant difference was observed between cohorts (*p* = 0.0325), with a higher pooled proportion in RCTs (89.93%; 95% CI: [39.07, 100.00]) compared with observational studies (30.48%; 95% CI: [13.89, 50.18]). High heterogeneity persisted across both subgroups (I^2^ > 98%). Random-effects model using REML estimator with Freeman–Tukey transformation [10,20,21,22,24,25,26,29,30].

Subgroup analysis demonstrated a significant difference in FGFR3 prevalence based on study design (*p* = 0.0325). The higher prevalence observed in randomized trials (89.9%) compared with observational studies (30.5%) reflects trial-level enrichment strategies rather than biological differences, confirming substantial selection bias (Figure 10).

#### 3.3.3. ROB Assessment

##### FGFR3

**Figure 11:**
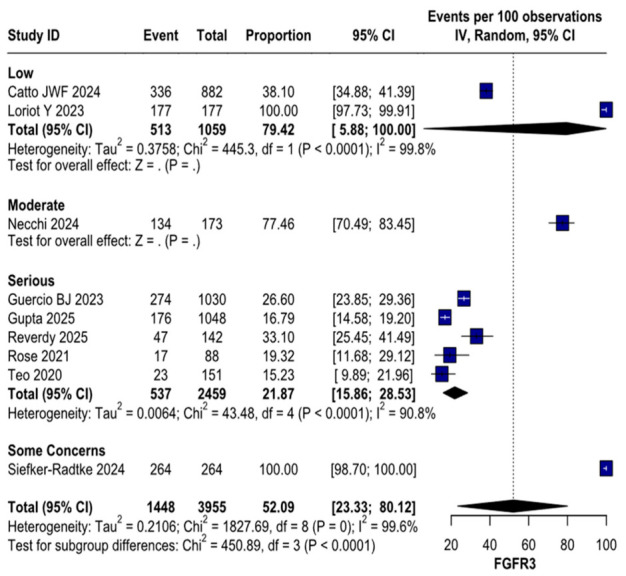
Subgroup analysis of FGFR3 prevalence by risk of bias (ROB). Significant differences were identified across ROB categories (*p* < 0.0001), with the lowest pooled proportion in studies with a serious risk of bias (21.87%; 95% CI: [15.86, 28.53]) and the highest in those with some concerns (100.00%) or low risk (79.42%). High heterogeneity (I^2^ > 90%) persisted across all multi-study subgroups. Random-effects model using REML estimator with Freeman–Tukey transformation [10,20,21,22,24,25,26,29,30].

Subgroup analysis based on ROB revealed significant differences in FGFR3 prevalence (*p* < 0.0001). The pooled proportion was lowest in studies with a serious ROB (21.87%; 95% CI: 15.86–28.53%), while studies categorized as low-risk and some concerns reported much higher rates of 79.42% and 100.00%, respectively. High heterogeneity (I2 > 90%) persisted across all multi-study subgroups (Figure 11).

##### Nectin-4

**Figure 12:**
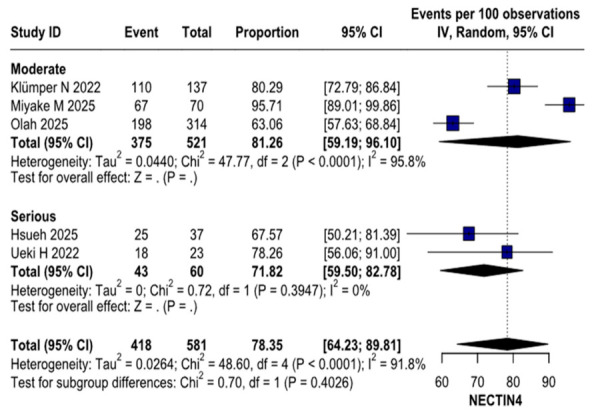
Subgroup analysis of nectin-4 prevalence by ROB. No significant difference was observed between categories (*p* = 0.4026), with pooled proportions of 81.26% (95% CI: [59.19, 96.10]) for moderate risk and 71.82% (95% CI: [59.50, 82.78]) for serious risk. Heterogeneity was high in the moderate-risk group (I2 = 95.8%) but absent in the serious-risk cohort (I2 = 0%). Random-effects model using REML estimator with Freeman–Tukey transformation [11,23,27,28,31].

Subgroup analysis by ROB showed no significant difference in nectin-4 prevalence (*p* = 0.4026). The pooled proportion was 81.26% (95% CI: 59.19–96.10%) in studies with moderate risk and 71.82% (95% CI: 59.50–82.78%) in those with serious risk. Heterogeneity was high in the moderate risk group (I^2^ = 95.8%) but absent in the serious-risk cohort (I^2^ = 0%) (Figure 12).

#### 3.3.4. Geographic Region

##### FGFR3

**Figure 13:**
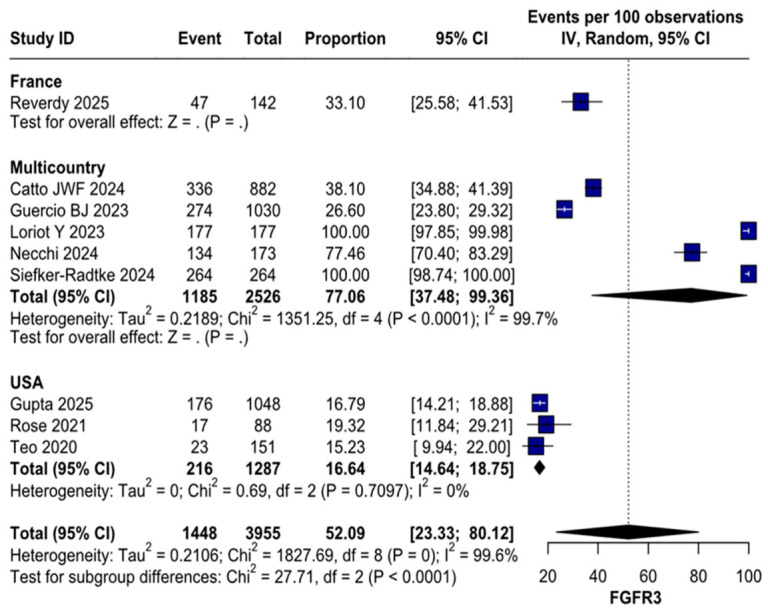
Subgroup analysis of FGFR3 prevalence by geographic region. Significant differences were observed across regions (*p* < 0.0001), with the highest pooled proportion in multicountry studies (77.06%; 95% CI: [37.48, 99.36]). Heterogeneity was absent in the USA subgroup (I^2^ = 0%) but remained high in the multicountry cohort (I^2^ = 99.7%). Random-effects model using REML estimator with Freeman–Tukey transformation [10,20,21,22,24,25,26,29,30].

Subgroup analysis by geographic region revealed significant differences in FGFR3 prevalence (*p* < 0.0001). The pooled proportion was highest in multicountry studies at 77.06% (95% CI: 37.48–99.36%), followed by France at 33.10% (95% CI: 25.58–41.53%) and the USA at 16.64% (95% CI: 14.64–18.75%). The USA subgroup showed no heterogeneity (I2 = 0%), whereas the multicountry group remained highly heterogeneous (I2 = 99.7%) (Figure 13).

##### Nectin-4

**Figure 14:**
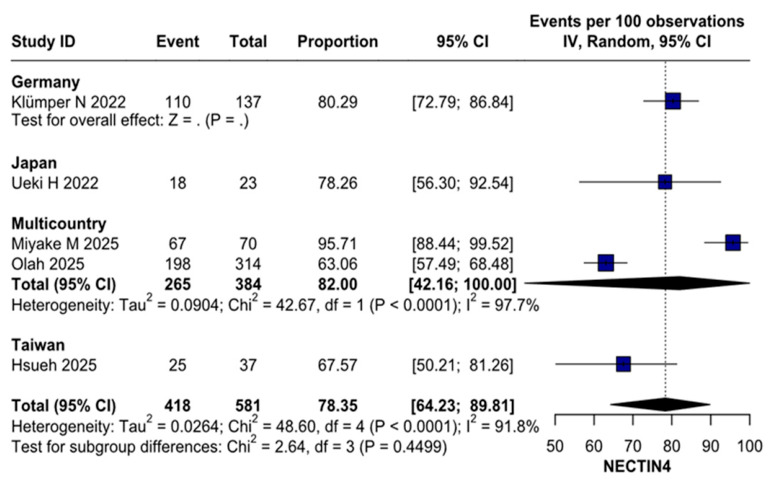
Subgroup analysis of nectin-4 prevalence by geographic region. No significant difference was observed across regions (*p* = 0.4499), with consistent pooled proportions in multicountry (82.00%; 95% CI: [42.16, 100.00), Germany (80.29%), Japan (78.26%), and Taiwan (67.57%) cohorts. High heterogeneity persisted overall (I^2^ = 91.8%), suggesting geographic origin is not a primary driver of nectin-4 expression variation. Random-effects model using REML estimator with Freeman–Tukey transformation [11,23,27,28,31].

The subgroup analysis by geographic region showed no significant difference in nectin-4 prevalence (*p* = 0.4499). The pooled proportions were relatively consistent across regions, with multicountry studies reporting 82.00% (95% CI: 42.16–100.00%), followed by Germany (80.29%), Japan (78.26%), and Taiwan (67.57%). High heterogeneity persisted overall (I2 = 91.8%), particularly within the multicountry subgroup (I2 = 97.7%), suggesting that geographic origin is not a primary driver of the variation in nectin-4 expression (Figure 14).

### 3.4. Quality Assessment

**Figure 15:**
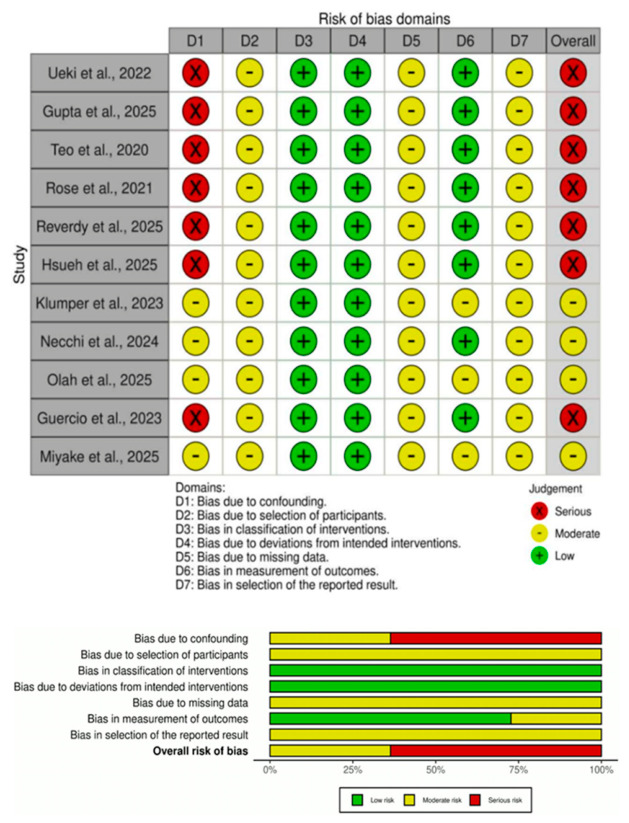
ROB assessment (ROBINS-I) summary for the observational studies [10,11,23,24,25,26,27,28,29,30,31].

**Figure 16:**
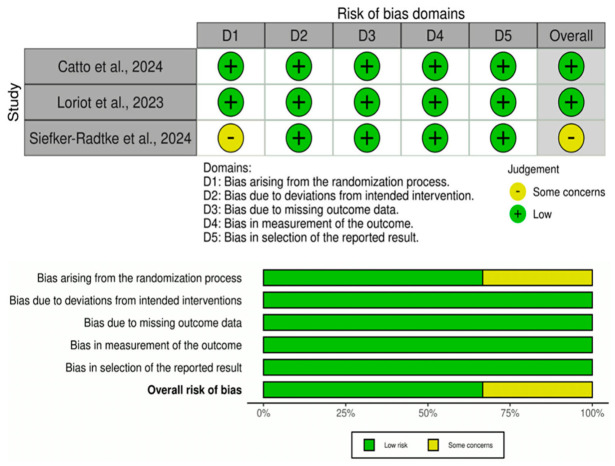
ROB assessment (RoB2) summary for the randomized studies [20,21,22].

**Figure 17:**
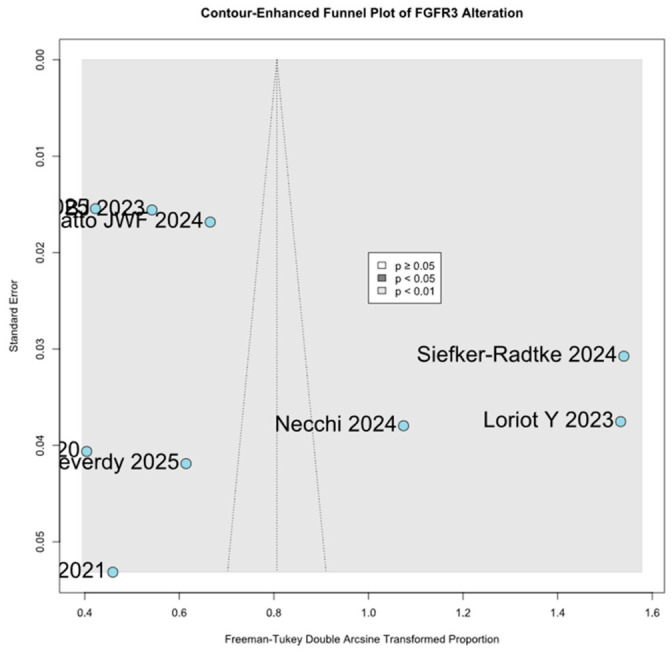
Contour-enhanced funnel plot for FGFR3 prevalence. The plot demonstrates visual asymmetry, suggesting the potential presence of publication bias among the included studies [10,20,21,22,24,25,26,29,30].

**Figure 18:**
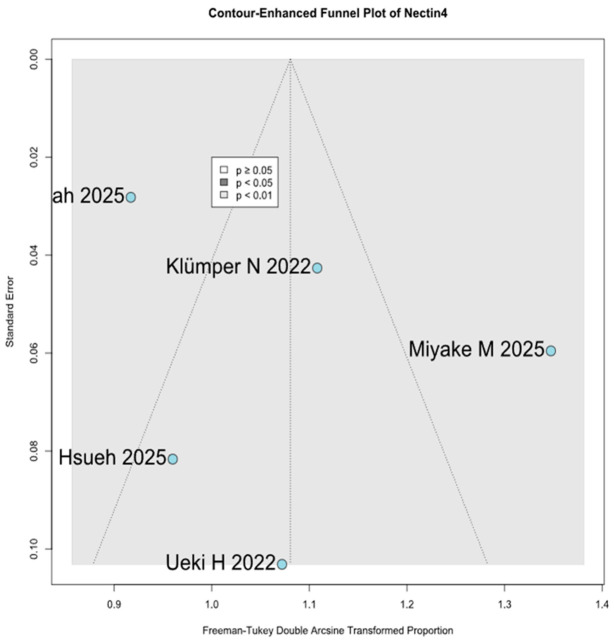
Contour-enhanced funnel plot for nectin-4 prevalence. The plot demonstrates visual asymmetry, suggesting the potential presence of publication bias among the included studies [11,23,27,28,31].

Of the eleven included observational studies, seven were assessed as having a serious risk of bias, and four as having a moderate risk of bias based on the ROBINS-I tool. The detailed evaluation is presented in Figure 15. Among the three included RCTs, one was assessed as having some concerns risk of bias, and two as having a low risk of bias based on the RoB2 tool. The detailed evaluation is presented in Figure 16. Publication bias was evaluated using contour-enhanced trim-and-fill funnel plot analyses, plotting individual study weights against point estimates. The funnel plots for the outcomes showed some asymmetry, indicating publication bias (Figure 17 and Figure 18).

## 4. Discussion

A key methodological limitation of this meta-analysis is the combination of biologically non-comparable populations, including biomarker-enriched clinical trials and unselected observational cohorts. This design inherently introduces selection bias, such that pooled estimates reflect study inclusion criteria rather than underlying disease biology. Given the extreme heterogeneity (I^2^ = 99% and 91%), pooled estimates should be interpreted descriptively rather than quantitatively, and subgroup patterns, rather than summary measures, should be emphasized. This analysis does not allow for conclusions regarding predictive value, treatment response, or optimal therapeutic sequencing, as these require comparative or patient-level data beyond the scope of a prevalence meta-analysis.

This systematic review and single-arm meta-analysis synthesizes the available evidence on two therapeutically actionable biomarkers in urothelial bladder cancer—FGFR3 alterations and nectin-4 expression—that have largely been investigated in separate translational and clinical research streams. The pooled estimates from 14 studies comprising 3955 patients indicate that both molecular features are common in urothelial malignancy: the pooled FGFR3 estimate (52%) and nectin-4 expression (78%) should not be interpreted as a true prevalence, but rather as a composite estimate influenced by study design, particularly enrichment strategies in clinical trials. These findings should be interpreted primarily as descriptive evidence of biomarker-reporting variability within the published literature and suggest that FGFR3 and nectin-4 should be interpreted as complementary, rather than independent, elements of the therapeutic landscape.

FGFR3 alterations represent one of the most thoroughly characterized molecular events in urothelial carcinoma. Their clinical relevance as a therapeutic target is now firmly established: the THOR trial demonstrated that erdafitinib significantly improved overall survival compared with chemotherapy (median: 12.1 versus 7.8 months; HR: 0.64) in patients with FGFR-altered metastatic urothelial carcinoma who had progressed on immune checkpoint inhibitors [22]. Pemigatinib similarly showed antitumor activity in previously treated patients with FGFR3 mutations or fusions, with objective response rates of approximately 18–23% in the FIGHT-201 trial [10]. Furthermore, in the non-muscle-invasive setting, erdafitinib prolonged recurrence-free survival compared with intravesical chemotherapy in BCG-treated high-risk patients harboring FGFR alterations (HR: 0.28; 95% CI: 0.1–0.6), thereby extending the relevance of FGFR-directed therapy beyond the metastatic space [20]. Our pooled prevalence estimate of 52% aligns with prior genomic landscape analyses, where FGFR3 somatic mutations have been reported in approximately 50% of all bladder cancers, with the frequency varying substantially by disease stage [32,33].

However, our pooled estimate should not be interpreted as a uniform biological constant across all disease states. The extreme heterogeneity observed (I^2^ = 99%) reflects genuine biological and methodological variability across the included studies. The classical molecular pathology literature has consistently associated FGFR3 alterations with luminal–papillary and non-muscle-invasive disease, with lower frequencies in muscle-invasive tumors. Large clinicogenomic data corroborate this gradient: Guercio et al. reported FGFR3 alterations in 39% of non-muscle-invasive tumors, 14% of muscle-invasive tumors, and 26% of distant metastases in a cohort of 1030 patients [30]. In the present analysis, the advanced/metastatic subgroup paradoxically yielded the highest pooled proportion (58.58%), a finding that most likely reflects case selection rather than a true reversal of disease biology—several metastatic cohorts and randomized trials were biomarker-enriched by design, particularly THOR and FIGHT-201, where FGFR3-altered status was a prerequisite for enrollment [10,21,22]. Meanwhile, observational studies sampled broader clinical populations, resulting in lower apparent frequencies. Accordingly, our results are best understood as a pooled prevalence within the published evidence base, not as an unbiased estimate of the natural distribution of FGFR3 across all disease stages.

Despite its biological importance, the predictive value of FGFR3 for response to systemic therapies remains uncertain. The data across studies are not entirely consistent. While some reports suggest reduced sensitivity to platinum-based chemotherapy, particularly in the neoadjuvant setting, others have failed to demonstrate meaningful differences in survival outcomes. In the multicenter retrospective IFUCA study, Reverdy et al. found no significant differences in progression-free survival (6.6 versus 7.5 months; HR: 1.27; *p* = 0.15), overall survival (22.1 versus 20.8 months), or objective response rates (70.7% versus 69.2%) between FGFR3-altered and wild-type tumors treated with first-line platinum-based chemotherapy [21]. Teo et al. had earlier reported that FGFR3-altered tumors may exhibit reduced sensitivity to platinum agents in selected settings, particularly in the neoadjuvant context, where lower pathological complete response rates were observed [24]. These conflicting findings suggest that the interaction between FGFR3 biology and chemosensitivity may be context-dependent, modulated by tumor stage, molecular subtype, and treatment setting rather than FGFR3 status alone.

The relationship between FGFR3 alterations and response to immune checkpoint inhibitors (ICIs) is equally complex. FGFR3-altered tumors are frequently associated with a non-T-cell-inflamed phenotype, which has been hypothesized to reduce immunotherapy responsiveness. However, clinical evidence has not consistently supported this hypothesis. Rose et al. demonstrated in a real-world cohort that FGFR3-altered and wild-type cancers were equally responsive to immune checkpoint blockade (12% versus 19%; *p* = 0.73), with equivalent T-cell receptor diversity despite the less inflamed microenvironment [26]. Similarly, Gupta et al. analyzed 819 patients with metastatic urothelial carcinoma and found no significant differences in real-world overall survival or progression-free survival between FGFR3-altered and wild-type patients treated with ICIs. However, they identified that when tumor mutational burden (TMB ≥10 mut/Mb) was combined with FGFR3 status, a more informative composite biomarker emerged, with FGFR3-altered patients trending toward longer survival [29]. The THOR trial cohort 2, comparing erdafitinib with pembrolizumab in anti-PD-(L)1-naive FGFR-altered patients, further complicated the picture: outcomes with pembrolizumab were better than anticipated (median OS: 11.1 months), comparable to those seen in non-FGFR-altered populations [21]. Collectively, these data indicate that FGFR3 is not simply a binary drug selection marker; it may function as a broader disease-state marker that intersects with immune contexture, lineage programs, and mutational burden. This complexity strongly argues for composite biomarker models rather than single-marker treatment algorithms.

In contrast with the stage-dependent variability seen with FGFR3, the nectin-4 findings in our analysis are more directionally consistent. Expression remained high across studies and did not differ significantly by tumor stage (*p* = 0.40) or geographic region (*p* = 0.45), suggesting that nectin-4 is a more stable therapeutic feature than FGFR3 in urothelial carcinoma. This finding is clinically significant because enfortumab vedotin (EV), the antibody–drug conjugate targeting nectin-4, has become a cornerstone of the treatment paradigm. The first-line standard of care for advanced urothelial carcinoma has been transformed following the EV-302 trial, which demonstrated a near doubling of overall survival with the combination of EV and pembrolizumab compared to conventional platinum-based chemotherapy (33.8 vs. 15.9 months; HR: 0.51). Long-term follow-up at 2.5 years confirms that these therapeutic responses remain durable over time [25,34]. Updated analyses with 2.5-year median follow-up have confirmed durable responses, with a median duration of response of approximately two years [35].

Despite a high pooled prevalence of 78%, the data reveal significant biological variability (I^2^ = 91%), which precludes viewing nectin-4 as a homogeneous marker. Research by Klümper et al. emphasizes that membranous expression levels frequently decline during metastatic progression, a phenomenon directly linked to the development of resistance to enfortumab vedotin [31]. Olah et al. demonstrated differential expression across histological and molecular subtypes, with the highest positivity rates in micropapillary (58%) and pure urothelial (30%) histologies and luminal subtypes (urothelial-like: 42%; genomically unstable: 34%), while basal (5%), mesenchymal (0%), and sarcomatoid (17%) tumors exhibited substantially lower expression [28]. Miyake et al. provided translational evidence that cytotoxic chemotherapy may downregulate nectin-4 through epithelial-to-mesenchymal transition, and that 43% of neoadjuvant chemotherapy-treated patients showed a luminal-to-basal subtype shift in cystectomy specimens [27]. These observations collectively suggest that a single archival specimen may not always provide an adequate representation of target expression at the time EV is being considered.

The predictive value of nectin-4 for platinum-based chemotherapy remains unsettled. Hsueh et al. reported a non-significant trend toward improved outcomes with higher membranous nectin-4 expression in patients treated with gemcitabine plus cisplatin (median PFS: 7.0 versus 4.0 months; adjusted *p* = 0.06), particularly in the cisplatin-based subgroup [23]. Conversely, Olah et al. found that patients with lower nectin-4 expression tended to derive more benefit from platinum-based chemotherapy in both adjuvant and neoadjuvant settings (*p* < 0.001 and *p* = 0.067, respectively) [28]. These seemingly contradictory results may reflect the complexity of nectin-4 biology: its expression correlates with luminal differentiation, which carries prognostic and predictive implications independent of the target protein. The interplay between molecular subtype, nectin-4 status, and chemosensitivity remains insufficiently disentangled and represents an important area for future investigation.

Evidence on the interaction between nectin-4 expression and immunotherapy response is limited but suggestive. Ueki et al. reported that strong nectin-4 expression in tumor cells was correlated with a significantly higher disease control rate (100%) in patients treated with pembrolizumab compared with lower expressors (50%), though the study was small (n = 23 with available tissue) and hypothesis-generating [11]. This association may reflect the broader relationship between luminal differentiation programs, nectin-4 expression, and immune microenvironment composition rather than a direct mechanistic link. Bahlinger et al. demonstrated that both Nectin-4 and TROP-2 were widely expressed in advanced urothelial carcinoma independently of FGFR3 alterations or PD-L1 expression, indicating that nectin-4 targeting may be broadly applicable regardless of immune biomarker status [36]. These findings further support the combined use of EV with pembrolizumab as implemented in EV-302.

This analysis does not include patient-level data and, therefore, does not allow evaluation of biomarker co-occurrence, correlation, or temporal evolution. A major strength of this review is that it places FGFR3 and nectin-4 within the same analytic frame, enabling consideration of their potential biological and therapeutic interplay. Biologically, both biomarkers are associated with luminal differentiation programs, yet they capture fundamentally different vulnerabilities: FGFR3 reflects an oncogenic signaling dependency exploitable by tyrosine kinase inhibitors, whereas nectin-4 represents a cell-surface target for antibody–drug conjugates. Emerging preclinical data suggest a direct mechanistic link between the two pathways. Clark-Garvey et al. demonstrated that FGFR3 inhibition with erdafitinib paradoxically upregulates nectin-4 protein expression in cell lines harboring FGFR3 alterations, an effect that appears to be mediated through the FGFR3/MEK signaling axis [20]. This upregulation was confirmed in both xenograft and syngeneic murine models, persisted even after erdafitinib withdrawal, and produced in vitro synergy between erdafitinib and enfortumab vedotin at lower doses of both agents [37]. Furthermore, NECTIN4 amplification has recently emerged as an independent genomic predictor of EV response: Klümper et al. reported that 96% of patients with NECTIN4-amplified metastatic urothelial carcinoma achieved objective responses to EV, compared with 32% in the non-amplified subgroup, with a 92% risk reduction for death in multivariable analysis [38]. These findings open a compelling translational avenue: sequential or combinatorial strategies leveraging FGFR inhibition to prime nectin-4 expression before ADC exposure. Although still preclinical, such rational drug sequencing based on molecular crosstalk represents a frontier in precision uro-oncology.

From a treatment sequencing perspective, FGFR3 and nectin-4 define partially overlapping but therapeutically distinct patient populations. Patients with FGFR3-altered disease may receive erdafitinib or pemigatinib following progression on immune checkpoint blockade, while nectin-4 expression supports the use of EV across a broader biomarker spectrum. With the advent of EV plus pembrolizumab as the first-line standard of care, the clinical question shifts from whether either biomarker matters to how they should be jointly operationalized when multiple active drug classes are available. However, the existing literature, including the studies in this meta-analysis, rarely report patient-level co-occurrence of FGFR3 and nectin-4, serial biomarker evolution under treatment pressure, or outcome-stratified analyses across sequential treatment lines. Filling this evidence gap is one of the most important translational priorities in advanced bladder cancer. Additionally, next-generation nectin-4-directed ADCs incorporating alternative payloads (such as topoisomerase 1 inhibitors) and novel constructs (including CAR-T cells targeting nectin-4) are in early-phase clinical development [39], further expanding the therapeutic repertoire and reinforcing the need for biomarker-informed patient selection. These observations are largely based on preclinical or early-phase data and should be considered hypothesis-generating rather than directly supported by this analysis.

This analysis also highlights critical methodological challenges in the biomarker literature. Assay harmonization remains poor for both biomarkers. FGFR3 positivity was variably defined across studies by mutation type, fusion, amplification, or trial eligibility criteria, and assessment methods ranged from next-generation sequencing to quantitative PCR. The European Association of Urology now recommends testing for FGFR3 alterations at the time of metastatic urothelial carcinoma diagnosis [40]. Moreover, emerging data suggest that artificial intelligence-based histopathological screening may enable rapid and cost-effective pre-screening of FGFR3 mutations from routine hematoxylin–eosin slides with high sensitivity (>93% in advanced/metastatic cases), potentially reducing the need for molecular testing by up to 40% [41]. For nectin-4, expression was assessed using immunohistochemistry with heterogeneous scoring systems, variable cut-off values, and no standardized minimum threshold required for ADC efficacy. Recent evidence suggests that NECTIN4 copy number amplification, detectable using fluorescence in situ hybridization, may offer a more stable genomic biomarker than protein expression via IHC, as gene-level amplification is subject to less spatial and temporal heterogeneity than membranous protein levels [38]. Future standardization of testing methodologies for both biomarkers will be essential to ensure reproducibility and clinical applicability.

This study has several limitations. The high level of heterogeneity across studies reflects both biological diversity and methodological variability, including differences in biomarker definitions and testing platforms. In addition, most of the available data are retrospective, which limits the strength of the conclusions. The analysis focused on biomarker prevalence rather than treatment effect; therefore, it does not provide comparative efficacy between therapeutic strategies.

FGFR3 alterations were heterogeneously defined across studies (mutations, fusions, amplification, and overexpression), and nectin-4 expression was assessed using non-standardized assays and thresholds. Pooling these biologically distinct entities likely contributed to heterogeneity and limits interpretability. Second, variability in biomarker assessment represents an important limitation: definitions of FGFR3 alterations differed across studies (mutations, fusions, amplifications, and overexpression), while nectin-4 expression was measured using heterogeneous immunohistochemical methods, scoring systems, and thresholds, limiting comparability. Third, tissue heterogeneity must be considered. Biomarker expression may differ between primary tumors and metastatic sites, particularly for nectin-4, where decreased expression during metastatic spread has been documented [28,31]. This raises important questions regarding the optimal timing and anatomical site of biomarker assessment. Fourth, the risk-of-bias profile was not trivial: most observational studies were judged to have serious or moderate risk of bias according to ROBINS-I, and funnel plot asymmetry raised the possibility of publication or small-study effects. Fifth, this review synthesized proportions rather than treatment effects; therefore, the findings should inform biomarker prevalence and translational framing but should not be overextended into claims of comparative efficacy between treatment classes. Sixth, most included studies were retrospective, and prospective validation remains limited, particularly for nectin-4 as a predictive biomarker. Seventh, this analysis was based on study-level data and did not include patient-level information, precluding evaluation of biomarker co-occurrence, correlation, and longitudinal evolution. Finally, the limited number of studies for nectin-4 (n = 5) restricts the robustness of pooled estimates and precludes reliable assessment of publication bias or subgroup effects.

These limitations are balanced by several strengths. This review was prospectively registered (PROSPERO: CRD420261309413), followed PRISMA 2020 guidelines, employed duplicate screening and extraction, and applied a random-effects framework with the restricted maximum-likelihood estimator. Sensitivity analyses indicated that no single study alone explained the pooled estimates; however, this does not overcome the substantial between-study heterogeneity. The consistency of these sensitivity analyses is reassuring in the literature, where individual high-profile trials or molecular cohorts can dominate clinical perception.

From a translational perspective, the findings highlight variability in reported biomarker prevalence and underscore the need for standardized methodologies and better-designed studies. In clinical practice, FGFR3 testing remains important for identifying candidates for FGFR-targeted therapy; however, this analysis does not establish treatment sequencing or comparative therapeutic benefit. For nectin-4, future studies should move beyond simple positivity thresholds and examine membrane localization, staining intensity, intratumoral heterogeneity, and concordance between primary and metastatic sites. Prospective cohorts should incorporate paired and longitudinal sampling, especially after chemotherapy, checkpoint blockade, or ADC exposure, as emerging data suggest that target expression may evolve under treatment pressure [27,31]. In parallel, clinical trials should incorporate biomarker-stratified sequencing analyses to determine which patients derive the greatest benefit from FGFR inhibition, EV-based combinations, or rational sequential strategies. The exploration of FGFR3 inhibitor-induced nectin-4 upregulation as a therapeutic priming strategy deserves dedicated clinical investigation [37]. In summary, FGFR3 and nectin-4 are both clinically relevant biomarkers in urothelial carcinoma, but they serve different purposes in practice. FGFR3 primarily identifies patients who may benefit from targeted therapy, whereas nectin-4 defines a broader population eligible for antibody–drug conjugates. Moving forward, efforts should be made to document their presence and understand how they should be used together within increasingly complex treatment pathways. Addressing this will likely require prospective, biomarker-driven studies that reflect real-world clinical sequencing rather than isolated therapeutic settings. A multimodal approach combining genomic, transcriptomic, and protein-level data—integrated with molecular subtype classification and immune contexture assessment—will likely be required to achieve truly personalized treatment strategies in bladder cancer.

## 5. Conclusions

This systematic review and single-arm meta-analysis highlights substantial variability in reported FGFR3 alterations and nectin-4 expression across bladder cancer studies. Due to extreme heterogeneity, selection bias from biomarker-enriched trials, and inconsistent biomarker definitions, pooled prevalence estimates should not be interpreted as precise biological frequencies. Rather, they should be viewed as descriptive summaries of reported frequencies across heterogeneous clinical and methodological contexts. Future research should prioritize standardized biomarker definitions, harmonized assay thresholds, and more homogeneous study designs to enable clinically meaningful interpretation.

## Data Availability

No new data were created or analyzed in this study. Data sharing is not applicable to this article.

## Abbreviations

The following abbreviations are used in this manuscript:

FGFR3	Fibroblast growth factor receptor 3
ICIs	Immune checkpoint inhibitors
PCR	Polymerase chain reaction
ADC	Antibody–drug conjugate
CAR-T	Chimeric antigen receptor T-cell therapy
EV	Enfortumab vedotin
ROB	Risk of bias
NMIBC	Non-muscle invasive bladder cancer
MIBC	Muscle-invasive bladder cancer
ROBINS-I	Risk of bias in non-randomized studies
REML	Restricted maximum likelihood
